# Research on the evolution of express packaging recycling strategy considering virtual incentives and heterogeneous subsidies

**DOI:** 10.1038/s41598-023-35543-4

**Published:** 2023-05-25

**Authors:** You Zhilong, Hou Guisheng

**Affiliations:** College of Economics and Management, Shandong University of Science and Technology, 579 Qianwangang Road, Huangdao District, Qingdao, Shandong China

**Keywords:** Environmental sciences, Environmental social sciences, Evolutionary theory

## Abstract

With the growth of e-commerce business volume, a large amount of express packaging waste is generated, causing certain damage to the environment. In response to this problem, the China Post Bureau pointed out the plan to strengthen express packaging recycling, and large e-commerce platforms such as JD.com have taken actions. Starting from this background, this paper uses a tripartite evolutionary game model to study the strategies evolution of consumers, e-commerce companies and e-commerce platforms. At the same time, the model considers the influence of platform virtual incentives and heterogeneous subsidies on equilibrium evolution. The study found that with the increase of virtual incentives from the platform to consumers, consumers converge to the strategy of "participating" in express packaging recycling faster and faster. When the assumption of participation constraints for cosumers is relaxed, the virtual incentives of the platform are still effective, but it will be affected by the initial willingness of consumers; when the e-commerce platform uses a single method to subsidize, it can effectively encourage e-commerce companies to use green packaging items. Compared with direct subsidize, the policy flexibility of the discount coefficient is stronger, in addition, moderate double subsidies can also achieve the same effect, and the e-commerce platform can make decisions based on the actual situation. The cyclical fluctuations in the strategies of consumers and e-commerce companies in the case of high additional profit coefficients of e-commerce companies may be the reason why the current express packaging recycling program is not effective. In addition, this article also discusses the influence of other parameters on the equilibrium evolution, and gives targeted countermeasures.

## Introduction

China News Service reported in 2019 that the total value of e-commerce transactions in China in 2018 surge to 31.63 trillion yuan, with an increase of 8.5% year-on-year. The business volume of express delivery services reached 50.71 billion, with a rising rate of 26.6% year on year. On June 21, 2021, the State Post Bureau released the latest data, during this year's 618 carnival period, the country's postal express industry received more than 6.59 billion express items, a year-on-year increase of 24.24% (real-time monitoring data from the State Post Bureau showed that by June 1, 2021), China's express delivery business volume had exceeded 40 billion. Various types of packaging materials have been developed and are widely used in order to prevent from being destroyed during transportation. Adding more colors, patterns and shapes factors into additional packaging has become a prevalence of the packaging strategy of enterprises. Relevant literature has pointed out that packaging attributes can affect the psychological perception of consumers and thus improve the market competitive power for enterprises^1–3^. With the continuous development of e-commerce and express business volume increasing year by year, a lot of the express package waste were generated, the dangers of packaging waste on the environment gradually aroused people's concern. Moreover, as the environmental protection consciousness and the concept of ecological civilization is becoming more and more popular, people begin to explore the new way express packaging recycling. At present, the phenomenon of excessive packaging and "nesting doll packaging" is particularly common in the express logistics industry. Under the current "double carbon background", it is of practical significance to realize the "slimness" and green transformation of express packaging. However, regrettably, as far as the current situation is concerned, the recycling rate of express packaging is still relatively low. People's Daily pointed out (August 11, 2020 07:45, source: People's Daily online-People's Daily. The original address: http://finance.people.com.cn/n1/2020/0811/c1004-31817533.html) it is estimated that China's current overall delivery packaging waste recovery rate poorly less than 10%, the recovery rate of which was below 20%. Even worse, padding and adhesive tape recovery rates was close to zero. To solve these problems, the State Post Bureau launched an implementation to promote green packaging in the express industry in 2016, encouraging manufacturers, consumers and other parties to make joint efforts to improve the recycling of express packaging, and strive to basically establish the express packaging recycling system by 2020. Major Chinese e-commerce platforms such as Taobao, JD.com and Suning have set some certain policies and practices related to anhancing the recycling of express packages and encouraged merchants and consumers to actively participate in them. However, limited by consumers' awareness of environmental protection and recycling costs, the acceptance and popularity of the recycling of express packaging in the downstream mode are not very high. Therefore, from the perspective of the participation of the three parties, this paper studies the virtual incentives provided by the e-commerce platform to consumers, and the evolution of the tripartite strategy under the circumstance that the e-commerce platform adopts different incentive modes for the e-commerce enterprises.

As far as the existing foreign literature is concerned, most of the studies focus on packaging recycling in a general sense, while few studies specifically focus on express packaging. For example, some foreign researchers study related issues from the perspective of packaging design, recycling system and recycling mode. Kondo et al.^4^ studied the reuse and recycling of various packages from the perspective of life cycle. Duhaime et al.^5^ studied the recyclable packaging utilization of Canada Post and large mailing customers. Stuart Ross and Evans et al.^6^ studied the recycling strategy of plastic packaging, while Pati et al.^7^, using the goal programming model, studied the recycling of paper packaging. In addition, some literatures have studied the greening and recycling of packaging from the perspective of reverse supply chain^8,9^. Klaiman et al.^10^ used discrete experiments and found that consumers' willingness to pay for packaging materials, he found that consumers' willingness to pay for plastic materials was positively correlated with the recyclability of packaging materials. With the concept of sharing economy gradually gaining popularity, some researchers began to study from the perspective of sharing packaging. Leite^11^ pointed out that recyclable packaging has good product protection, cost reduction and environmental benefits, and Silvas et al.^12^ demonstrated the benefits of shared packaging with an case study. However, some literatures have pointed out the disadvantages of shared packaging, such as high costs of transportation, cleaning and maintenance, storage and heavy capital investment^10^. In addition, there are also costs caused by loss and misplacement of packages^13^. Nevertheless, some researchers point out that the overall benefits of packaging recycling outweigh its disadvantages^14^. Twede and Clarke^15^ even point out that shared packaging is a global trend.

Other studies focused on the division of responsibility for packaging waste recycling or treatment. For example, SujitDas^13^ summarized three forms of joint collection by manufacturers, independent recycling by manufacturers, third-party recycling from the perspective of different recycling subjects. After studying relevant recycling policies, Wilmshurst et al.^16^ proposed the production responsibility extension system of “who produces, who is responsible”, and applied this practice to the formulation of packaging waste recycling. In addition, the literature summarizes and introduces the practice and experience of waste treatment in the UK, summarizes the management policies of the UK government into three types: sustainable development strategy, waste recycling and environmental protection action, and points out that some policy guidance can be used to encourage enterprises and the public to actively participate in it^6^. Mohamed Alwaeli^17^ studied the impact of product charging policies and relevant directions of the European Union on the recycling level of waste packaging in Poland. Selki^18^ pointed out that the design of green packaging system should take into account the protective effect of packaging for products and facilitate logistics distribution. Nuno Ferreira da Cruz et al.^19^ studied the recycling plans of Germany, France, Portugal and other European countries, and proposed interest coordination between the government and the industry for corresponding waste management under the extended industrial production responsibility system. Levine et al.^20^ analyzed and studied the financial problems of packaging waste from the perspective of the government. Green packaging is a kind of solid waste, the related research of municipal solid waste should also arouse our attention. Pitchayanin Sukholthaman et al.^21^ summarized the types and hazards of municipal solid waste. He also analyzed the effectiveness of current measures. This paper pointed out that the government should play a leading role in waste treatment. The methods and measures of the public and other institutions are the supplementations.

Some studies dealing with green supply chain are worthy of attention. While some studies have paid attention to carbon tax, carbon quota and carbon subsidy in this topic^22^. Wang et al. studied low-carbon supply chain contract design from the perspective of green supply chain network^23^. Guo et al. used the cooperative game method to study the carbon emission level and cost-sharing strategy in low-carbon supply chain^24^. Mandni et al. studied the pricing strategies of green products and non-green products, explored the competition between supply chains under the government-leading situation. Besides, they also gave the sensitivity analysis of green strategy, governance tariffs and other important variables in detail^25^. Fahimnia et al. constructed some flexible green supply chain models^26^. Luo et al. constructed a multi-stage game model considering different scenarios and explored the influence of equilibrium price, product greeness as well as after-sales service on supply chain^27^. Jiang et al. studied the strategy selection and interaction process of polluting enterprises, the central government together with local governments, which pointed out that government policies have significant impact on the strategy selection of relevant parties^28^. Jiahui Yang et al. used the differential game theory who the divide recycling model of express packaging into three types: government-led, market-driven and cooperation-driven. The research points out that cooperation-driven has the highest income, and compared with market-driven, government-driven can achieve a certain degree of Pareto improvement. The study also pointed out the effectiveness of subsidies^29^.

The literature points out that for the recovery and treatment of waste, the effect of only through the government is usually not particularly ideal^30^. Many studies have proposed to establish a system involving multiple actors to jointly handle these public affairs^31–33^. In recent years, many studies have investigated the impact and dynamic changes of external policies on relevant stakeholders from the framework of evolutionary game theory^34,35^. Tang et al. studied the difference between the analysis results of evolutionary game and Nash equilibrium involving two subjects, and further analyzed the competition among consumers, enterprises and government in green supply chain for emission reduction^36^. Xu et al. combined manufacturers and suppliers into a two-subject evolutionary game framework for analysis, and the study also pointed out the significant role of government regulation^37^. Zhu et al. constructed a supply chain system consisting of retailers and third-party recyclers. They analyzed the evolutionary stability strategies under the circumstances of government participation and non-participation. The study pointed out that when the government participated, the system could reach the ideal equilibrium state faster^38^. Jun Dong et al. combined prospect theory and evolutionary game theory to study the strategy selection and evolution of consumers, regulators and electricity sellers. Furthermore, they pointed out that subsidies from regulators can significantly promote the increase of dynamic pricing. After considering prospect theory, it is found that customers’ willingness to accept dynamic pricing is negatively correlated with their degree of risk aversion^39^. Mengyuan Li and Xin Gao used an evolutionary game model to study the implementation process of enterprises' green innovation technology and clarify that subsidizing enterprises is better than subsidizing banks. Plus, the effect of early punishment is better than that of subsidies^40^. Considering the complexity of subjects and factors involved in express packaging recycling, this paper uses a tripartite evolutionary game model to study the strategy choice, factors’ influences and evolution equilibrium of e-commerce companies, e-commerce platforms and consumers. These existing studies have given the foundation for this study.

Different from the previous literature, the possible innovations of this paper are: ① This paper takes into account the virtual incentives of e-commerce platforms that exist in reality to consumers. For example, in order to promote consumers to participate in express packaging recycling, the Jingdong platform has launched Jingdou rewards (other e-commerce platforms, such as Taobao Tmall, have also launched similar express packaging recycling actions in cooperation with Cainiao Posthouse, but in terms of duration and influence, JD has received more attention and lasted longer than them). This paper has more practical significance to incorporate this into the model; ② This paper considers the implementation of incentives by e-commerce platforms, and the existing research mostly focuses on the situation where the government provides subsidy incentives. Moreover, this paper divides the incentive methods into direct subsidy and commission rate discounts. The practical value lies in: this paper finds the applicability of two different policy instruments.

## Theoretical models

### Problem description

Three parties are considered in the context of express packaging recycling—consumers, e-commerce companies, and e-commerce platforms. For consumers, they can choose whether to participate in express packaging recycling. Consumers can get a certain sense of satisfaction by participating in express packaging recycling. In the case of virtual incentives from e-commerce platforms is absent, consumers need to target a trade-off in the “satisfaction” and cost. In the case of virtual incentives, consumers can obtain certain incentives, such as Jingdou rewards for packaging recycling from JD.com, and these rewards can be directly use like cash during shopping on the platform. For e-commerce companies, they can choose to use ordinary packaging or green packaging. As far as ordinary packaging is concerned, corrugated boxes and plastic express bags (mostly non-degradable) are the most widely used packaging materials at present.The main reason may be the cost of these materials is relatively low. However, plastic products are more harmful to the environment. Green packaging is now commonly used in degradable plastic bags, tape-free cartons, etc. The cost of these packaging materials is distinctly higher than that of ordinary packaging materials, so they are not widely used in reality. It is worth noting that ordinary Cartons can also be recycled and reused, so consumers can also participate in recycling when e-commerce companies use ordinary express packaging materials. For e-commerce platforms, they can choose to support express packaging recycling or not support. In reality, Taobao, Tmall, JD, etc. have given some express packaging reward programs, such as traffic push, virtual incentives, etc. Some of these incentives are for consumers, and some are for e-commerce companies. For e-commerce platforms, there are two main incentives for e-commerce companies: direct subsidies and commission discount coefficients for entry fees(a form of fixed commission rate). Generally speaking, the e-commerce platform’s condition for merchants to settle in is a one-time entry fee plus a sales commission model, and these two forms of subsidies just correspond to these two parts.

### Evolutionary game parameter settings and assumptions

This paper considers a tripartite evolutionary game model consisting of consumers, e-commerce companies and e-commerce platforms. The model satisfies the following assumptions before it is proposed:

#### **Assumption 1**

E-commerce platforms can choose to support or not support express packaging recycling through various means, etc. The probabilities of the two strategies are recorded as $x,1 - x$; consumers can choose to participate in the express packaging recycling plan or not participate, the probabilities of these two strategies are recorded as $y,1 - y$; e-commerce companies can choose to use green express packaging or ordinary express packaging, and the probabilities of these two strategies are recorded as $z,1 - z$.

#### **Assumption 2**

When an e-commerce platform chooses a support strategy, it will provide certain incentives to e-commerce companies and consumers. Remember that the virtual incentives provided by the e-commerce platform for consumers participating in green packaging recycling are $D$. Enterprises can be motivated by direct subsidies $F$ and the commission discount coefficient $\theta$ for merchants to settle in, and the cost of the e-commerce platform to provide support strategies is $C_{1}$, and the income that can be obtained at this time is $G_{1}$. When the e-commerce platform chooses the unsupported cost of $C_{2}$, the income at this time is $G_{2}$.

#### **Assumption 3**

When consumers choose to participate in packaging recycling, the cost they need to pay is $L_{1}$. when they choose not to participate in green packaging recycling, there is neither cost nor benefit, and they can also gain psychological satisfaction $S$ when participating in express packaging recycling, and there is $S > L_{1}$(this formula can be seen as consumer participation constraints).

#### **Assumption 4**

**Figure 1:**
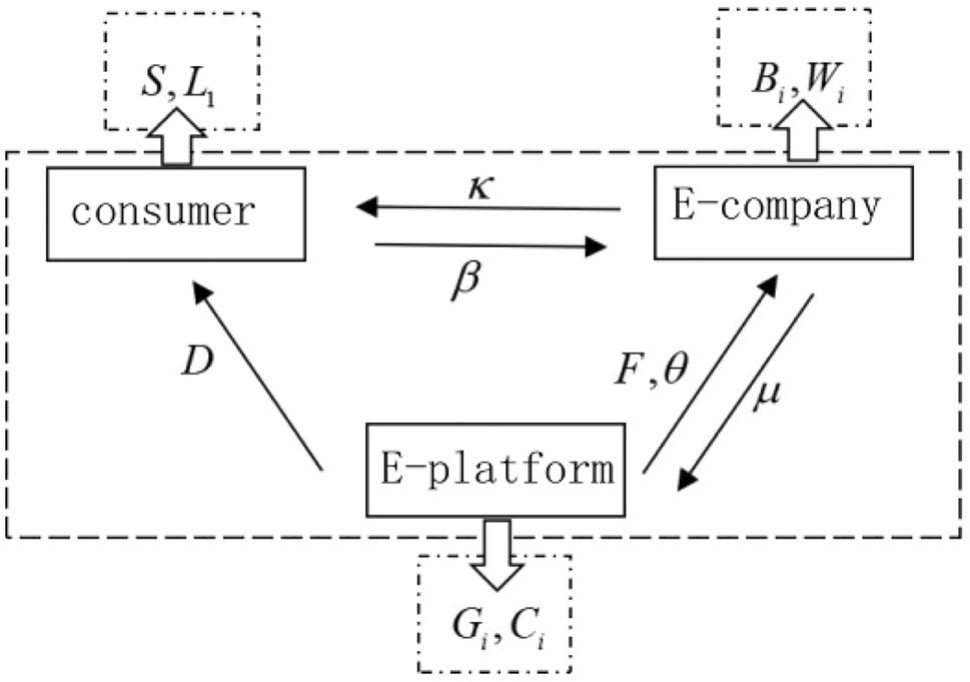
Parameter relationship diagram between subjects.

When an e-commerce company chooses to use green express packaging, the cost is $W_{1}$, the benefit is $B_{1}$, and the cost of using ordinary express packaging is $W_{2}$, and the benefit is $B_{2}$ and there is $W_{1} > W_{2}$. The basic commission coefficient for e-commerce companies to enter the e-commerce platform is $\mu$.when e-commerce companies choose to use green packaging and consumers participate in packaging recycling, they can obtain certain additional benefits (for example, consumers will help their publicity to improve their reputation, etc.), this benefit is positively related to the psychological satisfaction of consumer participation, denoted as $\beta S$. Since the cost of green express packaging is higher, this cost will be passed on to consumers with a coefficient of $\kappa$. The parameter settings and meanings are shown in Table 1 below. The schematic diagram of the relationship between the subjects is shown in Fig. 1 below.

**Table 1:** Parameter setting and meaning.

Parameter	Meaning	Parameter	Meaning
$x$	Probability/willingness of e-commerce platforms to support express packaging recycling	$B_{1}$	The benefits of e-commerce companies using green express packaging
$y$	Probability/willingness of consumers to participate in express packaging recycling programs	$B_{2}$	The benefits of e-commerce companies using ordinary express packaging
$z$	Probability/willingness of e-commerce companies to use green packaging	$W_{1}$	The cost of e-commerce companies using green express packaging
$D$	Virtual incentives provided by e-commerce platforms to consumers participating in recycling	$S$	Psychological satisfaction of consumers participating in express packaging recycling
$F$	E-commerce platform transfer payment subsidy for e-commerce enterprises	$W_{2}$	The cost of e-commerce companies using ordinary express packaging
$\theta$	Commission discount coefficient of e-commerce platforms for e-commerce companies	$L_{1}$	Costs for consumers to participate in packaging recycling
$C_{1}$	The cost of e-commerce platform to provide support	$\mu$	The basic commission coefficient for e-commerce companies to enter the e-commerce platform
$G_{1}$	The benefits of e-commerce platforms when they choose to support	$\kappa$	Pass-through factor for the cost of green express packaging(from E-company to consumer)
$C_{2}$	Costs when the e-commerce platform is not supported	$\beta$	The additional profit factor of e-commerce companies When e-commerce companies use green packaging and consumers participate in packaging recycling
$G_{2}$	Profits when the e-commerce platform does not support

### Return matrix and replication dynamic equation

According to the previous analysis and assumptions, the payoff matrix of the three-parties evolutionary game can be obtained as shown in Table 2 below:

**Table 2:** Summary of returns of three-party evolutionary games.

Strategy mix	E-commerce platform	Consumer	E-commerce enterprise
(support, join, use)	$\left\langle {G_{1} - C_{1} - D + (\mu - \theta )B_{1} - F} \right\rangle ,\left\langle {S - L_{1} + D - \kappa W_{1} } \right\rangle ,\left\langle {B_{1} - W_{1} - (\mu - \theta )B_{1} + F + \beta S} \right\rangle$		
(support, join, not use)	$\left\langle {G_{1} - C_{1} - D + \mu B_{2} } \right\rangle ,\left\langle {S - L_{1} + D} \right\rangle ,\left\langle {B_{2} - W_{2} - \mu B_{2} } \right\rangle$		
(support, not join, use)	$\left\langle {G_{1} - C_{1} + (\mu - \theta )B_{1} - F} \right\rangle ,\left\langle 0 \right\rangle ,\left\langle {B_{1} - W_{1} - (\mu - \theta )B_{1} + F} \right\rangle$		
(support, not join, not use)	$\left\langle {G_{1} - C_{1} + \mu B_{2} } \right\rangle ,\left\langle 0 \right\rangle ,\left\langle {B_{2} - W_{2} - \mu B_{2} } \right\rangle$		
(not support, join, use)	$\left\langle {G_{2} - C_{2} + \mu B_{1} } \right\rangle ,_{1} \left\langle {S - L_{1} - \kappa W} \right\rangle ,\left\langle {B_{1} - W_{1} - \mu B_{1} + \beta S} \right\rangle$		
(not support, join, not use)	$\left\langle {G_{2} - C_{2} + \mu B_{2} } \right\rangle ,\left\langle {S - L_{1} } \right\rangle ,\left\langle {B_{2} - W_{2} - \mu B_{2} } \right\rangle$		
(not support,not join, use)	$\left\langle {G_{2} - C_{2} + \mu B_{1} } \right\rangle ,\left\langle 0 \right\rangle ,\left\langle {B_{1} - W_{1} - \mu B_{1} } \right\rangle$		
(not support, not join, not use)	$\left\langle {G_{2} - C_{2} + \mu B_{2} } \right\rangle ,\left\langle 0 \right\rangle ,\left\langle {B_{2} - W_{2} - \mu B_{2} } \right\rangle$		

When the e-commerce platform chooses to support the strategy or not and the expected income of the strategy are recorded as $E_{11} ,E_{12}$, and the average income is recorded as $E_{x}$, there are:

1 $$\begin{aligned} E_{11} & = yz[G_{1} - C_{1} - D + (\mu - \theta )B_{1} - F] + y(1 - z)(G_{1} - C_{1} - D + \mu B_{2} ) \hfill \\ & \quad + (1 - y)z[G_{1} - C_{1} + (\mu - \theta )B_{1} - F] + (1 - y)(1 - z)(G_{1} - C_{1} + \mu B_{2} ) \hfill \\ & = G_{1} - C_{1} - yD - zF + z(\mu - \theta )B_{1} + (1 - z)\mu B_{2} \hfill \\ \end{aligned}$$

2 $$\begin{aligned} E_{12} & = yz(G_{2} - C_{2} + \mu B_{1} ) + y(1 - z)(G_{2} - C_{2} + \mu B_{2} ) \hfill \\ & \quad + (1 - y)z(G_{2} - C_{2} + \mu B_{1} ) + (1 - y)(1 - z)(G_{2} - C_{2} + \mu B_{2} ) \hfill \\ & = G_{2} - C_{2} + z\mu B_{1} + (1 - z)\mu B_{2} \hfill \\ \end{aligned}$$

3 $$\begin{aligned} E_{x} & = xE_{11} + (1 - x)E_{12} \hfill \\ & = x(G_{1} - G_{2} ) + x(C_{2} - C_{1} ) + G_{2} - C_{2} - xyD - xzF + zB_{1} (\mu - x\theta ) + (1 - z)\mu B_{2} \hfill \\ \end{aligned}$$

Similarly, when consumers choose to participate in green packaging recycling or not participate, the expected income are recorded as $E_{21} ,E_{22}$, and the average income is recorded as $E_{y}$:

4 $$\begin{aligned} E_{21} & = xz(S - L_{1} + D - \kappa W_{1} ) + x(1 - z)(S - L_{1} + D) + (1 - x)z(S - L_{1} - \kappa W_{1} ) \hfill \\ & \quad + (1 - x)(1 - z)(S - L_{1} ) = S - L_{1} + xD - z\kappa W_{1} \hfill \\ \end{aligned}$$

5 $$E_{22} = 0$$

6 $$E_{y} = yE_{21} + (1 - y)E_{22} = y(S - L_{1} + xD - z\kappa W_{1} )$$

In the same way, when an e-commerce company chooses to use green packaging materials or use ordinary packaging materials, the expected income are recorded as $E_{31} ,E_{32}$, and the average expected income is recorded as $E_{z}$:

7 $$\begin{aligned} E_{31} & = xy[B_{1} - W_{1} - (\mu - \theta )B_{1} + F + \beta S] + x(1 - y)[B_{1} - W_{1} - (\mu - \theta )B_{1} + F] \hfill \\ & \quad + (1 - x)y(B_{1} - W_{1} - \mu B_{1} + \beta S) + (1 - x)(1 - y)(B_{1} - W_{1} - \mu B_{1} ) \hfill \\ & = B_{1} - W_{1} + x[F - (\mu - \theta )B_{1} ] + y\beta S \hfill \\ \end{aligned}$$

8 $$\begin{aligned} E_{32} & = xy(B_{2} - W_{2} - \mu B_{2} ) + x(1 - y)(B_{2} - W_{2} - \mu B_{2} ) \hfill \\ & \quad + (1 - x)y(B_{2} - W_{2} - \mu B_{2} ) + (1 - x)(1 - y)(B_{2} - W_{2} - \mu B_{2} ) \hfill \\ & = B_{2} - W_{2} - \mu B_{2} \hfill \\ \end{aligned}$$

9 $$\begin{aligned} E_{z} & = zE_{31} + (1 - z)E_{32} \hfill \\ & = z(B_{1} - B_{2} ) + z(W_{2} - W_{1} ) + B_{2} - W_{2} + zx(F + B_{1} \theta - B_{1} \mu ) + zy\beta S - \mu B_{2} (1 - z) \hfill \\ \end{aligned}$$

According to the above analysis, the evolutionary game system composed of the above three replicating dynamic equations can be obtained as follows:

10 $$\begin{aligned} F(x) & = \frac{dx}{{dt}} = x(1 - x)(E_{11} - E_{12} ) \hfill \\ & = x(1 - x)[G_{1} - C_{1} - yD - zF - z\theta B_{1} + (1 - z)\mu B_{2} - G_{2} + C_{2} ] \hfill \\ \end{aligned}$$

11 $$F(y) = \frac{dy}{{dt}} = y(1 - y)(E_{21} - E_{22} ) = y(1 - y)(S - L_{1} + xD - z\kappa W_{1} )$$

12 $$\begin{aligned} F(z) & = \frac{dz}{{dt}} = z(1 - z)(E_{31} - E_{32} ) \hfill \\ & = z(1 - z)\{ B_{1} - W_{1} + x[F - (\mu - \theta )B_{1} ] + y\beta S - B_{2} + W_{2} + \mu B_{2} \} \hfill \\ \end{aligned}$$

## Model solutions

According to the opinions of Ritzberger and Weibull^41^, in the case of a three-way evolutionary game, only the pure strategy points need to be focused. According to Friedman’s view^42^, the stability of the differential equation system can be judged according to the Jacobi matrix. According to the replication dynamic equation of the three parties mentioned above, the Jacobi matrix can be obtained:

13 $$J = \left[ {\begin{array}{lll} \begin{gathered} (1 - 2x)[G_{1} - C_{1} - yD - zF - \hfill \\ z\theta B_{1} + (1 - z)\mu B_{2} - G_{2} + C_{2} ] \hfill \\ \end{gathered} & {x(1 - x)( - D)} & {x(1 - x)( - F - \theta B_{1} - \mu B_{2} )} \\ {y(1 - y)D} & {(1 - 2y)(S - L_{1} + xD - z\kappa W_{1} )} & {y(1 - y)( - \kappa W_{1} )} \\ {z(1 - z)[F - (\mu - \theta )B_{1} ]} & {z(1 - z)\beta S} & \begin{gathered} (1 - 2z)\{ B_{1} + x[F - (\mu - \theta )B_{1} ] \hfill \\ + y\beta S - W_{1} - B_{2} + W_{2} + \mu B_{2} \} \hfill \\ \end{gathered} \\ \end{array} } \right]$$

The eight pure strategy points of the evolutionary game model in this paper and their corresponding eigenvalues are shown in Table 3 below:

**Table 3:** Pure strategy points and their corresponding eigenvalues.

Equilibrium	The eigenvalues corresponding to the equilibrium point
$R_{1} (0,0,0)$	$G_{1} - C_{1} + \mu B_{2} - G_{2} + C_{2}$, $S - L_{1}$,$B_{1} - W_{1} - B_{2} + W_{2} + \mu B_{2}$
$R_{2} (0,0,1)$	$G_{1} - C_{1} - F - \theta B_{1} - G_{2} + C_{2}$, $S - L_{1} - \kappa W_{1}$,$- (B_{1} - W_{1} - B_{2} + W_{2} + \mu B_{2} )$
$R_{3} (0,1,0)$	$G_{1} - C_{1} - D + \mu B_{2} - G_{2} + C_{2}$, $- (S - L_{1} )$,$B_{1} + \beta S - W_{1} - B_{2} + W_{2} + \mu B_{2}$
$R_{4} (1,0,0)$	$G_{1} - C_{1} - D - F - \theta B_{1} - G_{2} + C_{2}$, $- (S - L_{1} - \kappa W_{1} )$,$- (B_{1} + \beta S - W_{1} - B_{2} + W_{2} + \mu B_{2} )$
$R_{5} (1,1,0)$	$- (G_{1} - C_{1} + \mu B_{2} - G_{2} + C_{2} )$, $S - L_{1} + D$,$B_{1} + [F - (\mu - \theta )B_{1} ] - W_{1} - B_{2} + W_{2} + \mu B_{2}$
$R_{6} (1,0,1)$	$- (G_{1} - C_{1} - F - \theta B_{1} - G_{2} + C_{2} )$, $S - L_{1} + D - \kappa W_{1}$,$- \{ B_{1} + [F - (\mu - \theta )B_{1} ] - W_{1} - B_{2} + W_{2} + \mu B_{2} \}$
$R_{7} (0,1,1)$	$- (G_{1} - C_{1} - D + \mu B_{2} - G_{2} + C_{2} )$, $- (S - L_{1} + D)$,$B_{1} + [F - (\mu - \theta )B_{1} ]{ + }\beta S - W_{1} - B_{2} + W_{2} + \mu B_{2}$
$R_{8} (1,1,1)$	$- (G_{1} - C_{1} - D - F - \theta B_{1} - G_{2} + C_{2} )$, $- (S - L_{1} + D - \kappa W_{1} )$,$- \{ B_{1} + [F - (\mu - \theta )B_{1} ]+ \beta S - W_{1} - B_{2} + W_{2} + \mu B_{2} \}$

According to the Lyapunov method^43^, when the eigenvalues corresponding to the equilibrium point are all negative, the point is asymptotically stable point. Combined with the relative size of the parameters in the known conditions above $S > L_{1} ,W_{1} > W_{2}$, it is easy to find that: $R_{1} ,R_{5}$ are unstable (because there are positive characteristics root $S - L_{1} + D > S - L_{1} > 0$). The positive or negative situations of the characteristic roots corresponding to other equilibrium points are more complicated. It is difficult to directly classify and discuss. Therefore, in order to explore the transformation of equilibrium points under different parameter changes more clearly and intuitively, the following sections will use numerical examples to explain different scenarios to discover more valuable management insights.

## Numerical simulations

In November 2019, JD Logistics launched the activity of “Environmental protection is knocking on the door”, and vigorously promoted the door-to-door recycling carton activity across the country. Consumers can hand over their idle express packaging cartons when JD.com is delivering express face to face. Consumers give it to the courier brother, and get a certain amount of Jingdou rewards (100 Jingdou equals 1 RMB). Currently, about 5 Jingdou are recycled for a small carton, and more for larger one. The commission coefficient of each e-commerce platform varies. The difference generally ranges from 5 to 30%. Based on the settings of the simulation parameters in the existing literature^24,27,44–47^, and meanwhile consider the real case of JD Mall, the simulating parameters of this paper are selected: $S = 2,\theta = 0.1,\beta = 0.2,u = 0.3,G_{1} = 3,G_{2} = 2,C_{1} = 1,C_{2} = 1,D = 0.5,F = 0.5,B_{1} = 3,B_{2} = 4,W_{2} = 1,W_{1} = 2,L_{1} = 1,\kappa = 0.1$. The reasons for parameter values chosen are as follows: $S$ represents the benefits of consumers' participation in packaging recycling, such as psychological satisfaction. Considering the constraints of behavior participation in reality, it is necessary to meet $S > L_{1}$ here. The setting here is mainly based on the reference^48^. The commission discount coefficient is one of the main characteristic parameters of this study, and the parameter variation range [0.1–0.5] is considered here. The parameter $\beta$ indicates that when consumers participate in green packaging, they can improve their recognition of e-commerce enterprises. Here, the parameter variation range is 0.2–0.8. In reality, the factor $u$ for e-commerce enterprises to settle down on the platform is 20–30%, such as Taobao and Tmall. The Xiamen Municipal Government has subsidized green packaging express enterprises in 2021. If the annual purchase cost of green packaging materials reaches more than 800,000 yuan, it will be subsidized by 10% of the annual purchase cost. The maximum subsidy amount is up to 200,000 yuan. These expenditures are the cost of funds supported by the government. Therefore, the range of parameter $F$ change [0.1–1] is considered here, On the other hand, the government's active participation in these activities that are conducive to the environment and ecology can convey a positive and responsible government image and improve its position in the hearts of consumers and enterprises. The characterization of this intangible benefit is expressed by parameters. Because it is difficult to find quantitative data in reality, the changes in the range of change^1–3^ are discussed here. The virtual incentives provided by platforms such as JD.com for consumers are often very small. On average, only 10–50 points can be exchanged for one package, which is only 0.1–0.5 RMB. However, considering the large user base of e-commerce platforms, the situation between the parameter change range [0.1–0.5] is investigated. In reality, the cost for e-commerce enterprises to choose green packaging is often 2–3 times or more than that of ordinary packaging, so set parameters $[(B_{2} - W_{2})/ (B_{1} - W_{1}] = 3$. As the Chinese saying goes, wool comes from sheep. The cost of e-commerce enterprises will ultimately be reflected in the price of products (including freight and express packaging fees), and these costs will ultimately be transfered to consumers. In order to find out the impact of the cost transfer coefficient on evolution, we will examine the situation when this parameter $\kappa$ is 0.2 and 0.5 respectively. At the same time, in order to make the analysis of the article more orderly, the simulation part of this article is divided into 3 subsections: “The virtual incentive changes of the platform to consumers affect the equilibrium evolution” section is the influence of the virtual incentive changes of the platform on consumers; “The evolution of the main body when the platform subsidy changes” section is the impact of changes for subsidy parameter (direct subsidy and commission discount coefficient) on the equilibrium; “Effects of other parameter changes on equilibrium evolution” section is the impact of other parameter changes on the equilibrium evolution. At the same time, to discuss the initial willingness^49^ of the three subjects vividly, we divide the initial willingness of the three subjects into three cases: {low, medium, high} = {0.2, 0.5, 0.8}.

### The virtual incentive changes of the platform to consumers affect the equilibrium evolution

**Figure 2:**
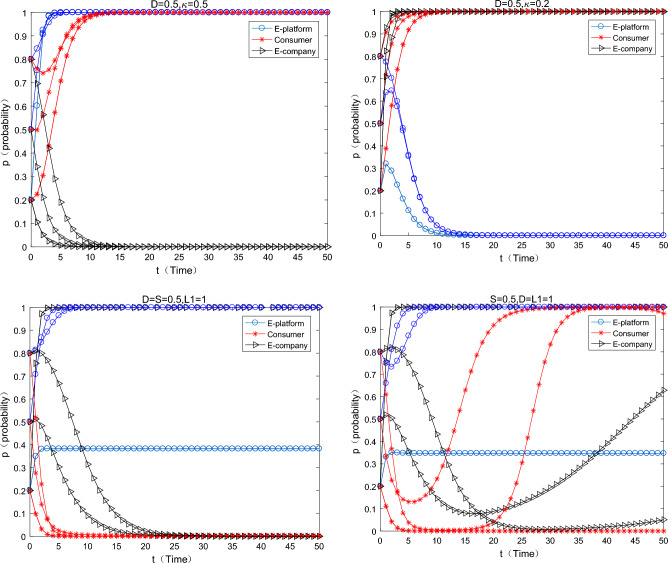
The influence of the virtual incentive change of the platform on the equilibrium evolution.

As can be seen from the Fig. 2 in the first row below, with the increase of virtual incentives for consumers by the platform, consumers converge to the strategy of “participating in” express packaging recycling faster and faster. But it is worth noting that it has been assumed $S > L_{1}$ in the assumptions that the consumer's participation constraint is positive, and virtual incentives can play a “icing on the cake” incentive effect at this time. A natural question is whether virtual incentives work if participation constraints are not satisfied. As can be seen from the graph in the second row, when the assumption of participation constraints is relaxed. That is, the benefit of consumers participating in express packaging recycling is less than the cost, at this time, with the increase of virtual incentives on the platform, consumers can be promoted to participate in express packaging recycling. What’s more, this conclusion is affected by the initial willingness of consumers. When the initial willingness to participate is relatively low (0.2), it is difficult for consumers to converge to 1 (participating in). When the initial willingness is medium to high (0.5, 0.8), consumers will eventually converge to 1 (participating in), but it will take a longer time. At this time, the probability of e-commerce companies using green express packaging is also very small, and they have all converged to the strategy of using “ordinary packaging”. In reality, although the JD platform implements the “Jingdou Rewards Program”, the overall recovery rate of express packaging is still relatively low. People’s Daily pointed out that the overall recovery rate of express packaging is less than 10%, and the simulation results are consistent with the typical fact.

### The evolution of the main body when the platform subsidy changes

#### The situation where the platform only uses payment transfer methods to subsidize

**Figure 3:**
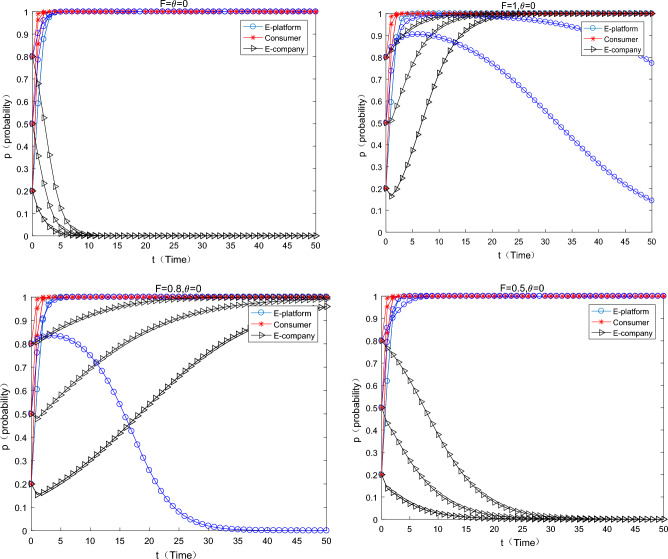
Only the impact of direct subsidies changes on equilibrium evolution.

As can be seen from the Fig. 3 below, when the platform only uses direct subsidies, with the increase of direct subsidies, the strategy of e-commerce companies has shifted from using “ordinary express packaging” to using “green express packaging”, which means that the direct subsidies are separately used can effectively motivate e-commerce companies to use green express packaging. However, with the increase of direct subsidies, the motivation of e-commerce platforms to choose direct subsidies is getting weaker and weaker, specifically when $F = 0.5 \Rightarrow F = 0.8$. when the platform’s direct subsidies have begun to work as an incentive for e-commerce companies, meanwhile the initial willingness of e-commerce platforms is medium or high level, the e-commerce platforms has the motivation to continue to subsidize. When the initial willingness of the e-commerce platform to choose support is relatively high, the motivation of the e-commerce company to make direct subsidies will drop sharply and converge to 0. At this time, consumers and e-commerce companies have a relatively strong awareness for green environmental protection, and the system will eventually converge to the strategy (no support, participate, green). When direct subsidies $F = 1$, e-commerce platform’s direct subsidies motivation is weaker, only when the initial willingness is very low (0.2), there is weak incentive in a very short period of time.

#### The platform only uses the commission discount coefficient to subsidize

**Figure 4:**
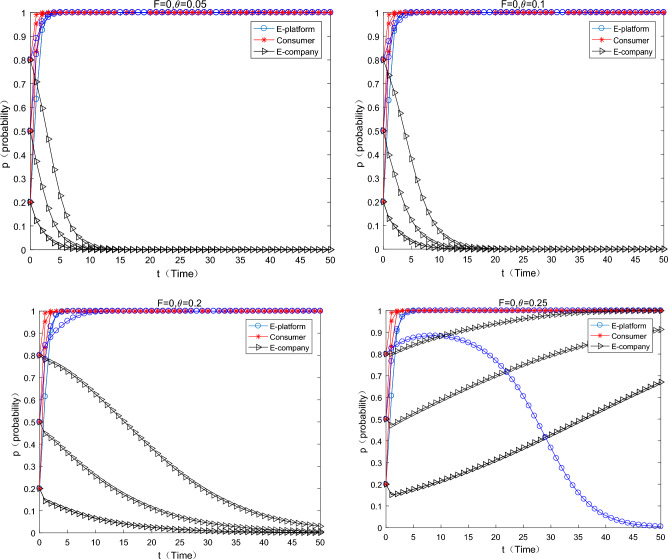
Effects on equilibrium evolution when discount coefficient changes only.

As can be seen from the Fig. 4 below, when the platform only uses the commission coefficient discount to subsidize the e-commerce enterprises, with the increase of the commission discount coefficient, the strategy of the e-commerce enterprise changes from using ordinary express packaging to using green express packaging. The incentive effect of the commission coefficient discounts is also significant. When it is $\theta { = }0.05 \Rightarrow \theta { = }0.25$,the incentive effect of the commission discount coefficient on e-commerce companies begins to work, especially when the initial willingness of e-commerce platforms transfer from low to medium (0.2–0.5). When the initial willingness of the e-commerce platform is high, the incentive of the e-commerce platform to choose commission discount subsidies is weak. At this time, both consumers and e-commerce enterprises have a strong awareness for environmental protection, and the system finally converges to (not support, participate in, green). Combined the previous findings, a comparison can be made $\frac{(0.25 - 0.05)}{{0.05}} \gg \frac{0.8 - 0.5}{{0.5}}$ [here, the concept of economic elasticity can be used to help elucidate. In fact, the denominator omits the concept of two formulas, such as the change of policy means and the change of strategic transition (the incentives start to work), which happens to be consistent with the concept of “elasticity” in economics], which means that the policy flexibility of the subsidies form of the commission coefficient discount is greater, the “tolerance” of the e-commerce platform is stronger, and the policy is more sustainable.

#### The situation where the platform uses both payment transfer and commission discounts

**Figure 5:**
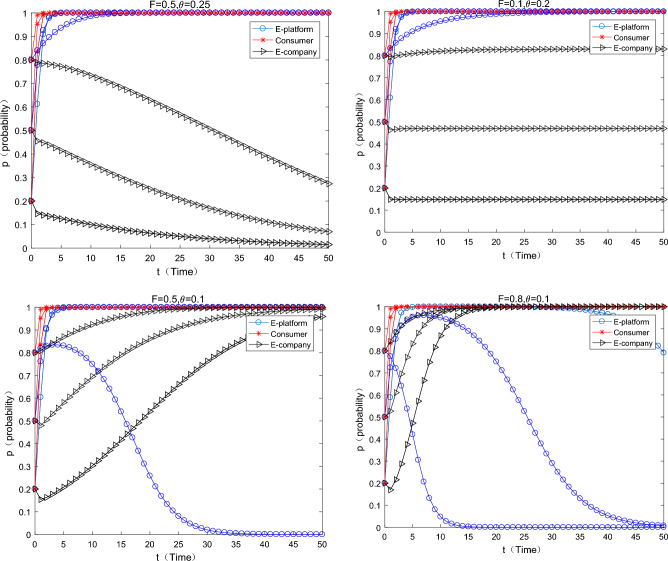
Effects of simultaneous changes on equilibrium evolution.

As can be seen from the Fig. 5 below, when the e-commerce platform uses both payment transfer and commission coefficients discount to subsidize e-commerce companies, as the two types of subsidies increase at the same time, the strategy choice of e-commerce companies is to choose “green packaging” strategy. Yet, when the direct subsidies rises to a certain level, the willingness to support the e-commerce platform will gradually decline, which is consistent with the situation when the payment transfer is used alone. It is worth noting that a moderate number for payment transfer and a lower commission discount coefficient ($F = 0.5,\theta = 0.1$) can achieve the incentive effect like a single-means subsidy ($F = 0,\theta = 0.25;F = 0.8,\theta = 0$). When both methods are used to subsidize, the results in all three cases are affected by the influence of the initial willingness of the e-commerce platform. When the initial willingness (to choose the strategy mix of triple 1) change from low to medium (0.2, 0.5), the e-commerce platform will eventually converge towards the “support” strategy. When the initial willingness is relatively high (0.8), the e-commerce platform will eventually will converge to the “not support” policy. Combined with the previous findings, e-commerce platforms can trim the sails on subsidy policies for e-commerce companies according to different scenarios.

### Effects of other parameter changes on equilibrium evolution

#### The impact of changes in the cost-transfer coefficient of e-commerce companies to users on equilibrium evolution

**Figure 6:**
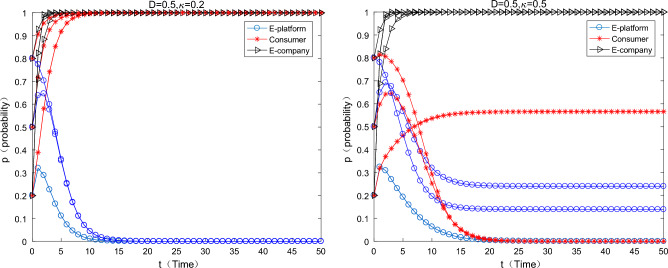
The influence of the change cost-transfer coefficient on the equilibrium evolution.

As can be seen from the above Fig. 6, as the cost-transfer coefficient of e-commerce companies to consumers increases, consumers will gradually change from “participation” strategy to “non-participation” strategy, e-commerce companies use green packaging. The proportion of the cost of express packaging passed on to consumers should not be too high, otherwise it will weaken the enthusiasm of consumers to participate in the greening of express packaging. China Youth Daily also pointed out that the cost of tape-free express cartons is 2–3 times more expensive than that of ordinary cartons in fact, while the cost of express packaging made of degradable plastic is 1.5 times that of ordinary plastic packaging. Therefore, the cost transmission coefficient should be carefully calculated.

#### The influence of the change of the additional income coefficient of e-commerce enterprises $\beta$ on the equilibrium evolution

**Figure 7:**
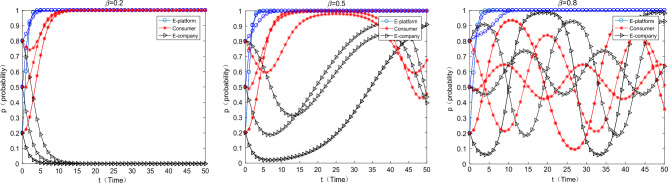
The influence of the change $\beta$ on the equilibrium evolution.

With the increase $\beta$ of e-commerce companies, the enthusiasm of e-commerce companies to use green packaging and the enthusiasm of consumers to participate in express packaging recycling will gradually increase. When it reaches a very high level ($\beta { = 0}{\text{.8}}$), the strategies of the two have cyclical fluctuations, but nor of them converges to 1. The meaning of this parameter $\beta$ is the coefficient of additional benefits brought by consumers’ satisfaction in participating in express packaging recycling when e-commerce companies that use green packaging. When the satisfaction of users participating in it is high, consumers more motivated to help e-commerce companies promote green express packaging, which will enhance the reputation of e-commerce companies and bring other intangible benefits. However, as this parameter continously increase, which shows that e-commerce companies are more “reliant” on consumers to improve their intangible benefits, and the motivation of e-commerce companies to choose green express packaging activities will weaker. Subsequently, consumers will reduce their participation. At this time, the “dependency” of e-commerce enterprises will decrease, which will increase their motivation to participate in the greening activities of express packaging. Therefore, the two are caught in a locked state of “trade-off”. It can also be found from the above Fig. 7 that when the willingness of consumers increases, the willingness of e-commerce companies decreases, and vice versa, the two always oscillate and diverge in such a state.

#### The impact of changes in the psychological satisfaction of consumers’ participation

**Figure 8:**
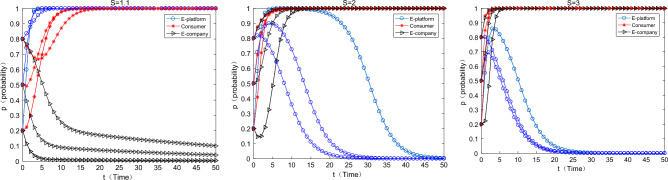
The impact of changes on $S$ for equilibrium evolution.

As can be seen from the Fig. 8 below, as the psychological satisfaction of consumers participating in express packaging recycling gradually increases, the strategic combination of e-commerce platforms, consumers and e-commerce enterprises gradually changes from (support, participate, not green) to (no support, participate, green). This suggests that by enhancing the satisfaction of consumer participation, we can effectively promote more consumers to participate in express packaging recycling and “enforce” e-commerce companies to choose green packaging. In reality, some offline publicity activities on the greening of express packaging and some small fun competitions on express packaging recycling can be held to enhance the satisfaction of consumers participating in it.

#### The impact of the cost of green express packaging on the equilibrium evolution

**Figure 9:**
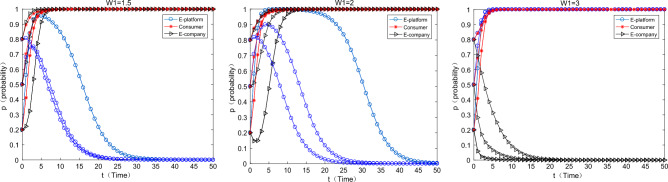
Impact of $W_{1}$ changes on equilibrium evolution.

As can be seen from the above Fig. 9, as the cost of green express packaging increases, e-commerce platforms, consumers, and e-commerce companies will converge to a strategic combination (not support, participate, green) → (support, participate, not green). At this time, the cost of green express packaging is too high, and the motivation for e-commerce companies to use them is very weak. In reality, the use of tape-free cartons and degradable plastic express packaging is not popular, because the cost of the two is much higher than that of ordinary cartons and ordinary plastic packaging. In the future, various types of green express packaging materials will be developed. For example, bio-based and starch-based degradable plastic bags can be used in the application of packaging materials and new methods to reduce pollution and carbon emissions. It's worth looking forward to reduce their costs through technological innovation.

#### The impact of changes in basic income supported by e-commerce platforms on equilibrium evolution

**Figure 10:**
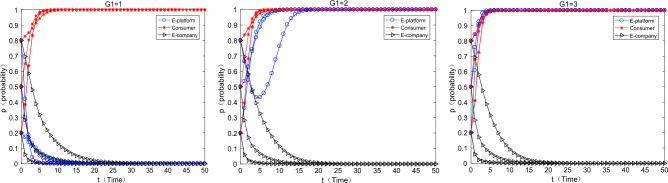
Impact of $G_{1}$ change on equilibrium evolution.

As can be seen from the above Fig. 10, as the revenue of the e-commerce platform’s choice of support increases, it gradually changes from the strategy of “not support” to “support”. In reality, relevant agencies such as the government and the media can praise relevant platforms for promoting the greening of express packaging, so as to enhance their reputation and brand effect or other intangible benefits, so as to increase their motivation for participation.

#### The impact of changes in the income of e-commerce companies choosing green express packaging on the equilibrium evolution

**Figure 11:**
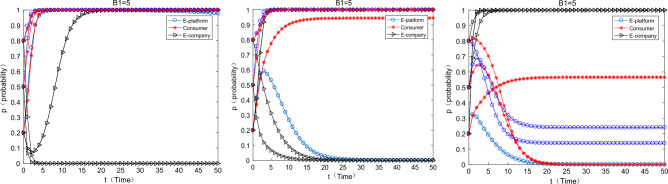
The impact of changes on equilibrium evolution.

As can be seen from the Fig. 11 below, as the income of e-commerce companies choosing green packaging increases, the strategy of e-commerce companies gradually changes from “non-green” to “green”, but the strategy of e-commerce platforms gradually changes from “support” to “no support”, the consumer’s strategy gradually changed from “participate” to “not participate”. This is because as the income of e-commerce enterprises increases, the commission income obtained by e-commerce platforms also gradually increases. At this time, the e-commerce platform has little motivation to support the express packaging recycling, so it will gradually choose “not support”. There is no virtual incentive for consumers, and consumers are less motivated to participate in express packaging recycling at this time.

The core findings and conclusions of the numerical simulation in this section are summarized in Table 4 below.

**Table 4:** Policy means and conclusions related to parameter changes.

Parameter change (policy instrument)	Main conclusion
$S > L_{1} \cap$ Virtual reward↑	The enthusiasm of consumers to participate in express recycling increased, and was not affected by the initial choice intention
$S < L_{1} \cap$ Virtual reward↑	Depends on the initial willingness to choose, media/high initial willingness to choose can increase the enthusiasm of consumers to participate in express packaging recycling
Single transfer subsidy↑	E-commerce enterprises can be encouraged to choose green express packaging, but the strategy is not sustainable
Commission factor discount for platform entry↓	It can encourage e-commerce enterprises to choose green express packaging, policy sustainability is strong
Mixed means (transfer subsidy↑ + commission factor discount ↓)	Moderate transfer payments and lower commission discounts can achieve similar incentive effects, and the policy sustainability is modest
Cost transfer coefficient↓	Consumers are more active in taking part in express package recycling
Additional income of e-commerce enterprises↑	The strategy appears oscillating instability phenomenon
Consumer intangible benefits/psychological satisfaction↑	The policy remains in the participating state
The cost of green express packaging↑	The enthusiasm of e-commerce enterprises to use green express packaging is reduced
Platform support revenue↑	Increased enthusiasm for platform support
Income from the use of green express packaging by e-commerce enterprises↑	Both platform support and consumer engagement are less motivated

## Discussion and conclusions

### Discussion

Through the above analysis, it can be seen that when consumers satisfy the participation constraints, setting virtual incentives on e-commerce platforms can promote consumers to participate in express packaging recycling. This viewpoint is consistent with other literatures^50^. Setting incentive mechanism can effectively change the strategy selection and evolution strategy of stakeholders. However, the question worth thinking about is whether incentives can be realized in other ways when participation constraints are not satisfied. It is found in the above that changing the initial choice intention of the subject can play a significant role. On the one hand, mobilization can be carried out through administrative means. For example, Shanghai put forward mandatory regulations on the sorting and recycling of urban waste, and then Beijing, Shenzhen and other places gradually followed. On the other hand, the initial willingness to choose is related to the environmental atmosphere of the whole society to a certain extent. If the atmosphere of the whole society to participate in environmental protection is strong, the public will have a higher initiative to participate in the recycling of express packaging. In reality, for example, the exchange ratio of Jingdou incentive and RMB adopted by JD is about 1:100. Overall, the incentive to participate in the recycling of express packaging is not very strong, so it has little effect in reality. At the same time, the subsidy of the platform can also be regarded as an incentive for e-commerce enterprises. For example, the e-commerce platform encourages e-commerce enterprises to choose more green express packaging by reducing the commission coefficient of e-commerce enterprises. In the analysis of this paper, we focus on two types of incentives: commission coefficient discount and direct subsidy(payment transfer). The study confirmed the motivational effects of the subsidy alone and both, but also found some interesting differentiated results. When e-commerce platform use only direct subsidy, the initial selection of willingness exert important influence, embodied in the means of direct subsidy in the initial choice of a relatively low, will play a particularly significant time is very short (converge to 1). However, with the growing increase of direct subsidy, e-commerce platform will gradually give up this kind of means. In other words, this approach is not very sustainable. On the other hand, the flexibility of the commission coefficient discount is greater. This finding is both interesting and economically intuitive. This is because the direct subsidy will forthrightly impact on income part of the e-commerce platform, and through the adjustment of commission coefficient discount, is essentially indirect, by influencing the e-commerce enterprises income part of the e-commerce platform, and the latter is more like “Without a sheep, there can be no wool ” (Chinese proverb), so the e-commerce platform more willing to adopt the latter one is natural. Another interesting finding is that the combination of the two may be better. A moderate level of direct subsidy and a relatively low initial willingness to choose can achieve the same effect as a large level of direct subsidy. This means that the e-commerce platform can flexibly choose the single form and combination form of these two ways.

On the other hand, the cost parameters has always been important which can influence the strategy selection and evolution of the subject under the paradigm of evolutionary game^51^. The cost transfer coefficient of e-commerce enterprises to express packaging will significantly affect the strategic choice of consumers. In reality, the cost of green express packaging (such as adhesive tape free) for e-commerce enterprises is 2–3 times higher than that of ordinary express packaging, while the cost of express bags made of degradable plastic is 1.5 times that of non-degradable plastic, and these costs will undoubtedly be transferred to consumers in some form ultimately. E-commerce enterprises in determining the cost transfer coefficient must be careful. In addition, if environmental publicity activities such as the use and recycling of green express packaging can improve the reputation of e-commerce enterprises, so as to improve their position in the market competition, the enthusiasm of e-commerce enterprises to use green express packaging will also be enhanced. Enterprises actively participate in the governance of social public affairs and actively assume responsibilities can help enterprises establish a good brand image^52^. Some studies also pointed out that consumers will prefer those enterprises with good image^53^, and the reputation benefits brought by a good corporate image can further increase the income of enterprise operation^54,55^. Findings in the numerical example indicates that this yield coefficient of ascension will increase consumers’ willingness to participate in. It can inhibit electricity enterprise’s desire to adopt green packaging to some extent, but too much is as bad as too little. High payment transfer may lead to cyclical shocks and bad situation, that partly explains why the present green courier packing in social acceptance and recognition is poor. For consumers, by improving the psychological satisfaction of using green packaging and participating in the recycling of green express packaging, they can be more effectively encouraged to participate in it. For example, consumers can be encouraged to participate in it by awarding knowledge contests, answering questions and setting certain prizes. With the notice of the fact that the cost of green express packaging materials is relatively high, more environment-friendly green materials can be developed through scientific and technological innovation in the future, and the use cost of all kinds of green materials can be reduced through innovation. The reputation mechanism has been proved in the previous literature in promoting the behavior choice^56^. For e-commerce platforms, reputation mechanism can also play a good effect. For example, its reputation can be improved by means of official media ’s praise. Such intangible benefits can have a positive effect on its market competitiveness.

After the research in this article, there are three additional points that need to be supplemented and further elaborated. These points are not the focus of the article, but they are still worth emphasizing and mentioning. At the same time, we will continue to focus on the related topics of express packaging recycling in future research from the following aspects.

① Firstly, the important role of subsidies in the recycling of express packaging cannot be ignored. Subsidies can stimulate the enthusiasm and initiative of entities to choose "environmentally friendly" strategies^57–60^. On the other hand, behaviors with public attributes such as environmental protection often do not have strong enthusiasm and sustainability if they rely heavily on the spontaneous actions of the public. The article points out this point, meanwhile, the findings are consistent with the conclusions in existing literature^61,62^. However, the use of subsidy tools can provide assistance to some extent in addressing this issue. Another aspect that closely related to subsidies is taxation, which constrains non recycling schemes (such as "non environmental-profitably strategies") through taxation. From another perspective, taxation has a "punitive" nature. For the issue of express packaging recycling, there are many entities involved (especially enterprise entities). In addition to the platforms and e-commerce enterprises we consider in the model, there are also many entities including logistics enterprises, Cainiao post stations that provide express pickup services (also belonging to similar commercial entities), express packaging production enterprises, and so on. How to design a tax scheme is a relatively complex issue, It is not only necessary to clarify who to pay the tax, but also to scientifically design tax rates.
② Secondly, it is necessary to consider some potential risks that may be implicit in waste management, especially in new scenarios. For example, using stronger plastic products or plastic materials as express packaging may trigger "plastic product pollution problems", while using multiple reusable express boxes (usually used for fresh products, such as the "green flow box" launched by the JD platform that can be recycled for more than 20 times) may cause food pollution or the spread of some bacteria due to inadequate disinfection. These problems can pose potential health risks to consumers. In these new recycling scenarios, although it may alleviate the excessive and pollution problems caused by packaging abuse, it has brought new risks, and the comprehensive weighing and consideration of different risk factors is also one of the issues that we need to consider in depth in the future.
③ Finally, due to the involvement of numerous stakeholders in the recycling issue of express packaging, we need to conduct a specific analysis based on the recycling scenario. Different stakeholders have different concerns and interests. We need to consider multiple factors as comprehensively as possible when formulating relevant policies. For example, for consumers, they need to pay some time and energy to participate in the recovery of express packaging, but they can gain some satisfaction. In combination with the enlightenment revealed by Richard Thaler's nudge theory^63^, We can set participating in express packaging recycling as the default option by E-software designing to encourage consumers to actively participate. For platforms, the intensity and quantity of virtual incentives can be moderately increased within the cost range. From the perspective of practical effect, the number of user participation attracted by virtual incentives is relatively limited, and more users with strong environmental awareness are actively participating. On the other hand, for e-commerce enterprises and business entities that were not addressed in this model, they should take a more active role in the recycling of express packaging. For e-commerce enterprises, more options and information on green express packaging should be provided on the shopping interface to help consumers better understand the positive effects of green express packaging on the environment and society. For logistics enterprises and packaging enterprises, they should also actively participate in the information sharing and innovative cooperation research for the development of new environmentally friendly express delivery materials, continuously improving the green degree of express packaging materials. For consumers, they should actively participate in the recycling of express packaging, learn more about the performance and related information of different express packaging materials, make reasonable decisions based on their own actual situation during future shopping time, and adopt more "environmentally friendly" strategies and behaviors. By comprehensively analyzing the interests and demands of different scenarios and entities, we can contribute more positively to the recycling of express packaging form a joint effort.

### Conclusions and implications

With the increasing volume of e-commerce transactions year by year, a large amount of express packaging waste has been generated. In the context of the current carbon neutrality goal being emphasized, consider how to promote e-commerce companies to choose green packaging and encourage consumers to participate in express packaging recycling programs and then accelerate the management of express packaging waste is a topic worthy of attention. This paper uses a tripartite evolutionary game model to study the strategy selection and evolution of e-commerce companies, e-commerce platforms, and consumers. At the same time, it considers the virtual incentives of e-commerce platforms to consumers and the heterogeneous subsidies of e-commerce platforms to e-commerce companies. The study found that with the increase of virtual incentives for consumers by the platform, consumers converged to the strategy of “participating in” express packaging recycling faster and faster. When the assumption of participation constraints is relaxed, the virtual incentive of the platform is still effective, but it will be affected by the initial willingness of consumers; the e-commerce platform adopts any single means to subsidize, which can effectively motivate e-commerce enterprises to use green packaging, but compared with direct subsidy, the coefficient discount is more likely to be used by e-commerce platforms for a long time, and the policy is more sustainable. In addition, moderate double subsidies can also achieve the same effect, and e-commerce platforms can make decisions based on the actual situation. When the value of certain parameters (when consumers participate in recycling and the e-commerce company chooses green packaging, the additional income coefficient obtained by the e-commerce company is large), the strategies of consumers and e-commerce companies are prone to shock and instability. This may be the reason for the ineffective implementation of the current express packaging recycling program. When e-commerce companies adopt green express packaging materials, the cost-transfer coefficient to consumers should not be too high. Improving the satisfaction of consumers participating in express packaging recycling can effectively promote more consumers to participate in express packaging recycling and encourage e-commerce companies to choose green packaging. The increase in the income of the e-commerce platform’s choice of support and the increase in the income of the e-commerce company’s choice of green packaging can also increase the willingness of the two to participate in express packaging recycling.

Based on the conclusions of this paper, the following policy recommendations are made:

1. E-commerce platforms can encourage consumers and e-commerce companies to participate in express packaging recycling through various means, such as establishing a “double points system” to praise consumers and e-commerce companies that perform well, and enhance the intangible benefits of both. Platforms can “trim the sails” when subsidizing e-commerce companies. For example, in the early stage, direct subsidies or double subsidies can be used to attract more “green” e-commerce companies to settle on the platform. Then gradually transitioned to the use of coefficients discount for subsidies. At the same time, e-commerce platforms can reduce the cost of consumer participation through door-to-door recycling and other methods. According to the view of Nobel Prize winner Richard Thaler, the probability of the main body to choose the prosocial behavior can be promoted through some boosting means that affect the cost, such as increasing the convenience of selecting the green packaging button in the platform software, and taking the green option as the default setting.
2. For e-commerce companies, when using high-cost green express packaging materials, the proportion of the cost passed on to consumers cannot be too high, otherwise it will dampen consumers’ willingness to participate, and the virtual incentives of the platform may also invalid. For e-commerce companies, they can cooperate with logistics companies and other institutions to manufacture new and environmental-friendly express packaging materials through R&D cooperation, and continuously reduce the cost of green express packaging. In addition, the total amount of express packaging waste can also be reduced by reusing express packaging. In addition, more e-commerce enterprises should be called upon to participate in the green express packaging plan. The recent green consumption points and other activities launched by the government can be linked up with the green express packaging plan to jointly contribute more to the cause of ecological and environmental protection.
3. For consumers, they can actively participate in express packaging recycling activities to help e-commerce companies that use green packaging materials. For themselves, when picking up the express package, it can directly deliver the express package to the courier for recycling. For example, JD.com has launched this method and encourages consumers to participate, which can reduce the cost of participating in the express package recycling. At the same time, consumers can reduce the total amount of express packaging waste through packaging sharing and reuse of packaging materials when sending express. Many reusable green express packaging materials have also been introduced abroad, such as 3 M Scotch ™ Flex&Seal, Boox, Loop, The box, goodFOOD, LogisALL, NGK, SuperJumbo, etc.Enhancing the use of express packaging can reduce packaging waste and packaging pollution.

## Data Availability

All data generated or analysed during this study are included in this published article.
